# Effect of Acupuncture on Cognitive Function of Insomnia Patients Compared with Drugs: A Protocol for Meta-analysis and Systematic Review

**DOI:** 10.1155/2021/6158275

**Published:** 2021-09-11

**Authors:** Shan Qin, Jing Jiang, Wen-Zhong Wu, Xiao-Qiu Wang, Han-Qing Xi, Qin-Qin Fang, Bin Xu, Cheng-Yong Liu

**Affiliations:** ^1^Jiangsu Province Hospital of Chinese Medicine, Affiliated Hospital of Nanjing University of Chinese Medicine, Nanjing 210029, China; ^2^NanJing University of Chinese Medicine, China

## Abstract

Insomnia, one of the most common sleep disorders, is thought to have an adverse effect on cognitive function. At the same time, people with cognitive dysfunction are more prone to insomnia. At present, pharmacotherapy is the main treatment for insomnia, but there are some shortcomings such as poor long-term efficacy and potential dependence. There is some evidence that acupuncture has some advantages in alleviating insomnia and improving cognitive function. This study is aimed at investigating the effects of acupuncture and drugs on cognitive function in patients with insomnia and evaluating the efficacy and safety of these two interventions, providing strong evidence for clinical decision-making. The study will retrieve eight major databases: China National Knowledge Infrastructure, Wanfang Database, VIP Database for Chinese Technical Periodicals, SinoMed, PubMed, Web of Science, Embase, and Cochrane Library. Dissertations, conference papers, and ongoing experiments will also be retrieved for supplement. Literature screening and data extraction will be completed by two authors independently (JJ and X-QW). If there were any disagreements, they would be discussed or referred to a third person for adjudication (W-ZW). Authors will use Cochrane risk of bias tool to assess the included studies. The Review Manager Statistical (RevMan) software is used to conduct the statistical process of meta-analysis, and funnel plot is used to evaluate reporting biases. The Grading of Recommendations Assessment Development and Evaluation (GRADE) Profiler can be used to be aware of the quality of evidence.

## 1. Introduction

Insomnia is one of the most common sleep disorders [1], which is mainly manifested as dissatisfaction with sleep time and quality, accompanied by daytime dysfunction, even if there is a good sleep environment and sleep opportunities [2]. One-third of the population in the United States is affected by insomnia, among which the elderly are the most common [3]. Meanwhile, compared with men, women are more prone to insomnia [4]. With the development of society, work pressure, and interpersonal conflict, the incidence of insomnia is still on the rise [5]. In addition to the health risks, insomnia also carries a substantial economic burden, costing society up to 100 billion dollars a year [6]. Studies have shown that insomnia can lead to cognitive function decline in patients through accumulation of amyloid-beta protein and increase of inflammatory factors [7]. They have deficits in attention, memory, and executive function. Furthermore, there is an increased risk of safety accidents and complications such as dysphagia and diabetes [8–11]. Insomnia and dementia influence each other. Dementia patients are more likely than the general population to suffer from insomnia, showing reduced sleep efficiency, increased nocturnal arousal, and early wakefulness [12]. There is still no way to completely cure dementia, but there are ways to prevent it by treating insomnia. Treatments for insomnia include psychotherapy, pharmacotherapy, and complementary and alternative therapies [13]. Among them, pharmacotherapy is the main way to treat insomnia at present [14]. The clinically commonly used sedative and hypnotic drugs are benzodiazepine receptor agonists, which are characterized by rapid absorption and quick action, but also have side effects such as “next day hangover” and potential dependence. At the same time, this drug can increase the risk of falling [15]. There are literatures which have been proved that acupuncture can effectively improve the quality of sleep and cognitive function [16–19]. It mainly improves the body's self-regulation ability and self-recovery ability by acting on different acupoints, to achieve the therapeutic effect [20], and has the advantages of easy operation, no adverse reactions, and extensive indications [21]. There is currently no evidence to prove the difference in efficacy and safety between acupuncture and drugs in cognitive function of insomnia patients. This meta-analysis is the first quantitative study to compare acupuncture with drugs from this perspective and to analyze whether acupuncture is effective for any type of insomnia.

## 2. Materials and Methods

This protocol was registered at PROSPERO and written based on the guideline of Preferred Reporting Items for Systematic Reviews and Meta-analyses Protocol (PRISMA-P) [22]. Details can be viewed in the Supplementary Table S1. PROSPERO registration number is CRD42021246046.

### 2.1. Criteria for Included Studies

#### 2.1.1. Types of Studies

The study will take into account all relevant randomized controlled trials. Language and publication status is not restricted. Studies that do not specify the method of randomization, but only mention randomization, also can be included. Blindness and allocation concealment are not confined. Clinical controlled trials also meet the requirements of the study and can be included.

#### 2.1.2. Types of Participants

Patients will be enrolled as long as they meet the diagnostic criteria for insomnia. There are no restrictions on gender, age, or nationality. Diagnostic criteria for insomnia are the following: International Statistical Classification of Diseases and Related Health Problems Version 10 (ICD-10) [23], Diagnostic and Statistical Manual of Mental Disorders Version 5 (DSM-5) [24], and International Classification of Sleep Disorder Version 3 (ICSD-3) [25]. Patients meeting other internationally recognized diagnostic criteria can also be included. If the diagnostic criteria are not clearly stated, the author will be contacted for further information [26]. Whether the patient is chronic insomnia or acute insomnia, simple insomnia or comorbid insomnia can be included.

#### 2.1.3. Types of Intervention

The experimental group received acupuncture as intervention measure, and any form of acupuncture could be included, including electroacupuncture, filiform needle therapy, body acupuncture, and auricular acupuncture. In acupuncture acupoints, the time and frequency of treatment are not limited. The control group was treated by drugs with insomnia treatment indications, including benzodiazepine receptor agonists, melatonin receptor agonists, antihistamine H1 receptor drugs, and orexin receptor antagonists. Studies using sham acupuncture in control groups will not be included. Myocardial infarction, diabetes, Parkinson's disease, and some other diseases are closely related to the decline of cognitive function [27–29]. The growth of age also affects people's cognitive function, so elderly insomnia and comorbid insomnia are more prone to cognitive decline [30]. The study will allow the elderly with underlying diseases and comorbid insomnia patients to use basic drugs, but the control group and the experimental group must be used at the same time.

#### 2.1.4. Types of Outcomes

The primary outcome indicators are the Montreal Cognitive Assessment Scale (MOCA) [31]. Secondary outcome indicators are Pittsburgh Sleep Quality Index (PSQI), Insomnia Severity Index Scale (ISI) [32, 33], and the incidence of adverse events.

#### 2.1.5. Exclusion Criteria

Research has critical data missing, and data cannot be obtained. Research that uses wrong randomized methods will be excluded (such as generating random sequences based on parity of birth dates); combination therapy should be excluded (for example, studies using acupuncture combined with cognitive behavioral therapy). Studies with a sample size less than 30 cases should be excluded.

### 2.2. Search Strategy

#### 2.2.1. Electronic Searches

Eight databases will be retrieved: China National Knowledge Infrastructure, Wanfang Database, VIP Database for Chinese Technical Periodicals, SinoMed, PubMed, Web of Science, Embase, and Cochrane Library. Retrieval time is from database inception to March 1, 2021. MeSH (Medical Subject Headings) and free text (title and abstract) will be combined to search. These MESHs will be included: acupuncture, needling, acupuncture and moxibustion, acupuncture therapy, electroacupuncture, cognition, cognitive function, insomnia, sleep initiation and maintenance disorders, randomized controlled trial, trial, randomly, randomized, and rct. The Chinese database will use the Chinese translation of the above search terms. The retrieval process of Embase can be seen in the Supplementary Table S2.

#### 2.2.2. Other Resources

Data from unpublished randomized controlled trials is available on the International Clinical Trials Registry, ClinicalTrials.gov, and Chinese Clinical Trial Registry. In addition, we will retrieve conference papers and dissertations to ensure that all randomized controlled trials that meet the criteria are retrieved to the maximum extent possible.

### 2.3. Data Collection and Analysis

#### 2.3.1. Selection of Studies

**Figure 1:**
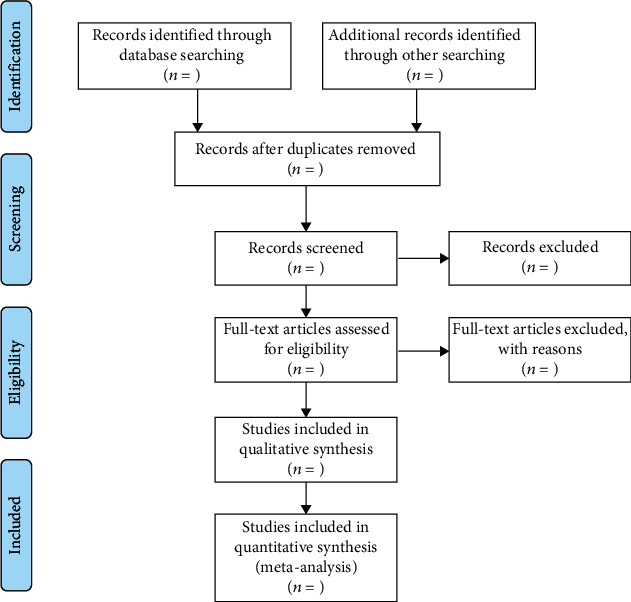


Literature retrieval and screening will be conducted independently by two authors (H-QX and Q-QF). First of all, duplicate literatures will be excluded, and a preliminary screening is conducted according to the title and abstract. Then, the literature that meets the standards will be screened by reading the full text. If there is a disagreement, the literature should be discussed or submitted to a third person (W-ZW) for evaluation. The EndNote software is used for literature management, and the reasons for exclusion should be recorded for excluded studies.  Figure 1 shows the process of literature screening in this research.

#### 2.3.2. Data Extraction

Two authors (JJ and X-QW) will independently produce a table to complete the data extraction work. The data extraction form includes the first author's name, publication year, follow-up situation, sample size, intervention measures, outcome, allocation concealment, randomization, selective reporting, blind method, integrity of outcome data, and the characteristics of the subjects (age, gender, course of disease, and educational level). If the results of the data extraction diverge, they are discussed or referred to a third person (W-ZW) for adjudication.

#### 2.3.3. Assessment of Risk of Bias in the Included Studies

This study will appraise the risk of bias according to the Cochrane Handbook bias risk assessment tool [34]. The risks of bias include selection bias, performance bias, detection bias, attrition bias, and reporting bias. The study will be classified into low risk of bias, high risk of bias, and unclear risk of bias. The two authors (H-QX and Q-QF) make the assessment, and if there is a disagreement, they discuss it or refer it to a third person (W-ZW).

#### 2.3.4. Dealing with Missing Data

If the required data is lacking, the author of the article will be contacted for relevant information, and if the data is still not available, the study with missing data will be excluded.

#### 2.3.5. Assessment of Heterogeneity

Heterogeneity is divided into clinical heterogeneity, methodological heterogeneity, and statistical heterogeneity. The clinical and methodological heterogeneity of the included studies is first assessed, and if the clinical heterogeneity is excessive, subgroup analysis will be performed based on clinical characteristics. *Q* statistic and *I*^2^ statistic are used to complete the statistical heterogeneity assessment [35]. *P* ≥ 0.1 and *I*^2^ ≤ 25% indicate low heterogeneity. *P* < 0.1 and *I*^2^ > 50% indicate high heterogeneity. The source of heterogeneity should be carefully analyzed when *I*^2^ ≥ 75.

#### 2.3.6. Assessment of Reporting Biases

If more than ten articles are included [36], the existence of reporting biases could be evaluated by the symmetry of the funnel plot.

#### 2.3.7. Data Synthesis

The RevMan software will be used for data processing. The effect index of the continuity variables is SMD (standardized mean difference) with 95% CIs (95% confidence intervals), and the categorical variable is RR (relative risk) and with 95% CIs, both of which are analyzed by inverse square examination. Considering the potential heterogeneity among acupuncture studies, this study will put to use the random effects model. If the heterogeneity is too large to identify its possible source, only descriptive analysis will be performed [37].

#### 2.3.8. Subgroup Analysis

If the included studies show obvious clinical heterogeneity, subgroup analysis will be conducted according to clinical characteristics. In this study, we will conduct subgroup analysis according to the gender and age of patients, the types of insomnia (such as simple insomnia/comorbid insomnia), acupuncture points, treatment duration and frequency, and acupuncture methods (such as electroacupuncture/filiform needle).

#### 2.3.9. Sensitivity Analysis

This study will carry out sensitivity analysis by changing the effect indicators, statistical model, and deleting each included study one by one [38] to verify the stability of the study results. If different conclusions are reached, the results of the meta-analysis are carefully obtained by discussion between the two authors (SQ and W-ZW) or by evaluation by a third person (X-QW).

### 2.4. Evidence Quality Evaluation

Cochrane Handbook bias risk assessment tool and GRADE Profiler are used to evaluate the quality of evidence by evaluating the factors that reduce the quality of evidence (risk of bias, inconsistency, indirectness, imprecision, publication bias) and the factors that increase the quality of evidence (large effect value, confounding factors that reduce efficacy, dose-effect relation) [39]. Evidence quality can be divided into four grades: high, medium, low, and very low [40].

## 3. Discussion

The adverse effects of insomnia and cognitive decline have caused a considerable burden on individuals, families, and society, while the incidence of insomnia and dementia continues to rise. Correct measures for patients with insomnia help to avoid the continuous deterioration of the situation. Therefore, we urgently need a relevant meta-analysis to provide reliable evidence for medical decision-making. There have been meta-analyses on acupuncture for insomnia, but they only focused on the effects of acupuncture on sleep and did not study from the perspective of acupuncture improving cognitive function in patients with insomnia [41–45]. Different from some studies only included in patients with comorbid insomnia [41–43]; this study as long as patients meet the diagnostic criteria of insomnia, whether simple insomnia or comorbid insomnia, can be included. Although it may increase the heterogeneity of the study, we can analyze whether acupuncture is effective for any type of insomnia. This study mainly includes four aspects: determining the inclusion and exclusion criteria and retrieval strategies, screening out all eligible randomized controlled trials at home and abroad, data extraction, data analysis, and processing. The study will also strive to obtain reliable conclusions through heterogeneity test, subgroup analysis, and sensitivity analysis. In addition, this study collected the relevant data of adverse events and compared the safety of acupuncture and drugs while understanding their efficacy. However, this study also has limitations: by comparing acupuncture with drugs alone, we cannot prove which is the best choice among all treatment options for improving cognitive function in insomnia patients. If the study does not include enough high-quality randomized controlled trials, the results of meta-analysis may be affected. The electronic databases of countries such as Korea and Japan will not be searched, which may lead to the existence of selective bias [46]. Different acupuncture methods may increase the heterogeneity of the study, but we will conduct a subgroup analysis to address this issue. In addition to insomnia, cognitive decline is associated with a variety of causes, including age and neurodegenerative disease, and there is no way to determine whether the decline in cognitive function in the included patients is due to insomnia.

## 4. Conclusion

This is a protocol for systematic review and meta-analysis. It mainly describes the specific methods for conducting the research, aiming to make the evidence provided by meta-analysis more powerful and accurate.

## Data Availability

The data analyzed and used to prepare this study are available from the corresponding author upon rational request.

## Supplementary Materials

Supplementary Materials Table S1: PRISMA-P Checklist. This protocol was written based on the guideline of Preferred Reporting Items for Systematic Reviews and Meta-analyses Protocol (PRISMA-P), and Supplementary Table S1 shows the details of checklist. Table S2: search strategy: EMBASE. Details of EMBASE's Search strategy can be seen in the Supplementary Table S2.
