# Supplementary material for: Effect of Acupuncture on Cognitive Function of Insomnia Patients Compared with Drugs: A Protocol for Meta-analysis and Systematic Review
Source: Behav Neurol. 2021 Sep 11;2021:6158275. doi: 10.1155/2021/6158275 (PMC8452430; doi:10.1155/2021/6158275)
Supplement: Supplementary Materials — Table S1: PRISMA-P Checklist. This protocol was written based on the guideline of Preferred Reporting Items for Systematic Reviews and Meta-analyses Protocol (PRISMA-P), and Supplementary Table S1 shows the details of checklist. Table S2: search strategy: EMBASE. Details of EMBASE's Search strategy can be seen in the Supplementary Table S2. [file 6158275.f1.pdf]

Effect of acupuncture on cognitive function of insomnia patients compared with drugs：A protocol for meta-analysis and systematic review

Supplementary Appendix

Effect of acupuncture on cognitive function of insomnia patients compared with drugs：A protocol for meta-analysis and systematic review

Cheng-Yong Liu[1]，Jing Jiang[1]，Xiao-Qiu Wang[1]，Han-Qing Xi[1]，Qin-Qin Fang[1] , Shan Qin[1]，Wen-Zhong Wu[1]

Table S1. PRISMA-P Checklist……………………………………………………….2-3

Table S2. Search strategy: EMBASE ………………………………………………………………...4-5

Table S1. PRISMA-P Checklist

Section and topic	Item	Checklist item	Page
ADMINISTRATIVE INFORMATION			
Title:			
Identification	1a	Identify the report as a protocol of a systematic review	1
Update	1b	If the protocol is for an update of a previous systematic review, identify as such	NA
Registration	2	If registered, provide the name of the registry (such as PROSPERO) and registration number	2
Authors:			
Contact	3a	Provide name, institutional affiliation, e-mail address of all protocol authors; provide physical mailing address of corresponding author	1
Contributions	3b	Describe contributions of protocol authors and identify the guarantor of the review	8
Amendments	4	If the protocol represents an amendment of a previously completed or published protocol, identify as such and list changes; otherwise, state plan for documenting important protocol amendments	NA
Support:			
Sources	5a	Indicate sources of financial or other support for the review	7
Sponsor	5b	Provide name for the review funder and/or sponsor	7
Role of sponsor or funder	5c	Describe roles of funder(s), sponsor(s), and/or institution(s), if any, in developing the protocol	7
INTRODUCTION			
Rationale	6	Describe the rationale for the review in the context of what is already known	2
Objectives	7	Provide an explicit statement of the question(s) the review will address with reference to participants, interventions, comparators, and outcomes (PICO)	2
METHODS			
Eligibility criteria	8	Specify the study characteristics (such as PICO, study design, setting, time frame) and report characteristics (such as years considered, language, publication status) to be used as criteria for eligibility for the review	3-4

Information sources	9	Describe all intended information sources (such as electronic databases, contact with study authors, trial registers or other grey literature sources) with planned dates of coverage	4
Search strategy	10	Present draft of search strategy to be used for at least one electronic database, including planned limits, such that it could be repeated	(Supplementary Table S2).
Study records:			
Data management	11a	Describe the mechanism(s) that will be used to manage records and data throughout the review	5-6
Selection process	11b	State the process that will be used for selecting studies (such as two independent reviewers) through each phase of the review (that is,screening, eligibility and inclusion in meta-analysis)	3-4
Data collection process	11c	Describe planned method of extracting data from reports (such as piloting forms, done independently, in duplicate), any processes for obtaining and confirming data from investigators	5
Data items	12	List and define all variables for which data will be sought (such as PICO items, funding sources), any pre-planned data assumptions and simplifications	3-6
Outcomes and prioritization	13	List and define all outcomes for which data will be sought, including prioritization of main and additional outcomes, with rationale	3
Risk of bias in individual studies	14	Describe anticipated methods for assessing risk of bias of individual studies, including whether this will be done at the outcome or study level, or both; state how this information will be used in data synthesis	5
Data synthesis	15a	Describe criteria under which study data will be quantitatively synthesised	5- 6
	15b	If data are appropriate for quantitative synthesis, describe planned summary measures, methods of handling data and methods of combining data from studies, including consistency (such as I^2, Kendall's τ)	5-6
	15c	Describe any proposed additional analyses (such as sensitivity or subgroup analyses, meta-regression)	5-6
	15d	If quantitative synthesis is not appropriate, describe the type of summary planned	5-6
Meta-bias(es)	16	Specify any planned assessment of meta-bias(es) (such as publication bias across studies, selective reporting within studies)	5
Confidence in cumulative evidence	17	Describe how the strength of the body of evidence will be assessed (such as GRADE)	6

Table S2. Search Strategy :EMBASE

#1 'acupuncture'/exp
#2 needling:ab,ti
#3 'acupuncture and moxibustion':ab,ti
#4 'acupuncture therapy':ab,ti
#5 electroacupuncture:ab,ti
#6 #1OR#2OR#3OR#4OR#5
#7 'sleep initiation and maintenance disorders':ab,ti
#8 'disorders of initiating and maintaining sleep':ab,ti
#9 dims:ab,ti#10 'early awakening':ab,ti
#11 'awakening, early':ab,ti
#12 'nonorganic insomnia':ab,ti
#13 'insomnia, nonorganic':ab,ti
#14 'primary insomnia':ab,ti
#15 'insomnia, primary':ab,ti
#16 'transient insomnia':ab,ti
#17 'insomnia, transient':ab,ti
#18 'rebound insomnia':ab,ti
#19 'insomnia, rebound':ab,ti
#20 'secondary insomnia':ab,ti
#21 'insomnia, secondary':ab,ti
#22 'sleep initiation dysfunction';ab,ti
#23 'dysfunction, sleep initiation':ab,ti
#24 'dysfunctions, sleep initiation':ab,ti
#25 'sleep initiation dysfunctions':ab,ti
#26 'insomnia':ab,ti
#27 'insomnia disorder':ab,ti

#28	'insomnia disorders':ab,ti
#29	'insomnias':ab,ti
#30	'chronic insomnia':ab,ti
#31	'insomnia, chronic';ab,ti
#32	'psychophysiological insomnia':ab,ti
#33	'insomnia, psychophysiological':ab,ti
#34	'insomnia'/exp
#35	#7 OR #8 OR #9 OR #10 OR #11 OR #12 OR #13 OR #14 OR #15 OR #16 OR #17 OR #18 OR #19 OR #20 OR #21 OR #22 OR #23 OR #24 OR #25 OR #26 OR #27 OR #28 OR #29 OR #30 0R #31 OR #32 OR #33 OR #34
#36	'cognition'/exp
#37	cognitions:ab,ti
#38	'cognitive function';ab,ti
#39	'cognitive functions';ab,ti
#40	'function, cognitive':ab,ti
#41	'functions, cognitive':ab,ti
#42	#36 OR #37 OR #38 OR#39 OR #40 OR #41
#43	#6 AND #35 AND #42
#44	'randomized controlled trial':ab,ti
#45	trial':ab,ti
#46	'randomly':ab,ti
#47	'randomized':ti,ab
#48	rct:ti,ab
#49	#44 OR #45 OR #46 OR #47 OR #48
#50	#6 AND #35 AND #42 AND #49
